# Artificial Intelligence in Perioperative Medicine: A Proposed Common Language With Applications to FDA-Approved Devices

**DOI:** 10.3389/fdgth.2022.872675

**Published:** 2022-04-25

**Authors:** Ryan L. Melvin, Matthew G. Broyles, Elizabeth W. Duggan, Sonia John, Andrew D. Smith, Dan E. Berkowitz

**Affiliations:** ^1^Department of Anesthesiology and Perioperative Medicine, University of Alabama at Birmingham, Birmingham, AL, United States; ^2^Department of Radiology, University of Alabama at Birmingham, Birmingham, AL, United States

**Keywords:** artificial intelligence, AI, machine learning, algorithm, FDA approval

## Abstract

As implementation of artificial intelligence grows more prevalent in perioperative medicine, a clinician's ability to distinguish differentiating aspects of these algorithms is critical. There are currently numerous marketing and technical terms to describe these algorithms with little standardization. Additionally, the need to communicate with algorithm developers is paramount to actualize effective and practical implementation. Of particular interest in these discussions is the extent to which the output or predictions of algorithms and tools are understandable by medical practitioners. This work proposes a simple nomenclature that is intelligible to both clinicians and developers for quickly describing the interpretability of model results. There are three high-level categories: *transparent, translucent*, and *opaque*. To demonstrate the applicability and utility of this terminology, these terms were applied to the artificial intelligence and machine-learning-based products that have gained Food and Drug Administration approval. During this review and categorization process, 22 algorithms were found with perioperative utility (in a database of 70 total algorithms), and 12 of these had publicly available citations. The primary aim of this work is to establish a common nomenclature that will expedite and simplify descriptions of algorithm requirements from clinicians to developers and explanations of appropriate model use and limitations from developers to clinicians.

## Introduction

The list of medical uses for Artificial Intelligence (AI) and Machine Learning (ML) is expanding rapidly (1). Recently, this trend has been particularly true for anesthesiology and perioperative medicine (2, 3). Deriving utility from these algorithms requires medical practitioners and their support staff to sift through a deluge of technical and marketing terms (3). This paper provides an aid for separating the signal of utility from the noise of jargon.

This work proposes a straightforward nomenclature for describing the interpretability and appropriate use of AI/ML products that will be intuitive to developers and clinicians alike. The applicability and utility of this terminology of these terms is then applied to Food and Drug Administration (FDA) approved AI/ML algorithms (1) with perioperative utility. Such a standardized language may speed discussion and understanding among technical developers and clinical users. To this end, there are three standardized terms for conveying interpretability and indicating the appropriate use of systems and products based on AI/ML. The terms are *transparent, translucent*, and *opaque*.

*Opaque* describes a system that (a) estimates non-linearly applied parameters that require advanced analysis (external to the product itself) to be understood, (b) estimates such a large set of parameters that a human cannot interpret them unless aided by a tool external to the product, or (c) provides a prediction with no indication as to the reason for the prediction. *Translucent* describes a product that incorporates non-specific methods for assisting the end user in understanding possible reasons for a prediction that would otherwise be categorized as “opaque.” *Transparent* describes a product that estimates a linearly applied parameter or relatively small set of parameters and is implemented to indicate to the user how much each feature influences the output or indicates the rationale for a prediction (e.g., providing exact weights for features considered). Alternatively, a transparent system's prediction is easily verifiable at the time the prediction is made using information the system provides.

For example, consider a clinician wanting to predict future glucose values for a patient (4). A careful developer for such a model would be concerned with several factors including whether the clinician desires simply a prediction (opaque) or a prediction with an explanation (translucent or transparent). If an explanation is needed, the developer may wonder how specific to each patient the explanation needs to be. Would a list of factors considered be sufficient (translucent), or does the clinician need to know exactly how much each factor contributed to the prediction for each patient (transparent)?

This imagined developer seeks to understand the level of interpretability required for this algorithm. However, there is also a tradeoff that the developer is considering. Requiring more interpretability limits the types of algorithms that can be used. The deep learning algorithms that currently automate driving, image recognition, and recently folded protein structure estimation (among many other things) tend to lack interpretability (opaque). On the other end of the spectrum, generalized linear models (such as logistic regression) tend toward high interpretability (transparent) but often have less accuracy than deep learning algorithms.

The situation also works in reverse. Consider a developer attempting to explain a new model to a randomly selected clinician. The clinician may wonder how an algorithm works or why it makes certain predictions. For some products, such questions are easy to answer (transparent). For others, it is exceedingly difficult (opaque). And the distinction between such systems at times seems arbitrary. Rather than developers and clinicians continuously engaging in this discussion *de novo* for every project and collaboration, presented here are three standardized terms for conveying the interpretability and indicating the appropriate use of AI/ML models—*transparent, translucent*, and *opaque*.

These ideas behind the terms are not new (5–7). Phrases such as “glass box,” (8) “white box,” (9) “gray box,” (10) “interpretable,” (11) “explainable,” (6, 8, 11–15) and “black box” (8, 10–14) are often used to describe the complexity of algorithms from a somewhat technical perspective. Proposed here are less technically intended terms meant to describe the perspective of the end user rather than the developer—a distinction discussed later with several examples. In addition to being first and foremost clinician-friendly, these terms are intended to convey sufficient technical information to developers for understanding the types of algorithms appropriate for the desired use case. Note that these terms do not describe the underlying mathematics of an algorithm or even the technical details of a particular implementation; rather, they describe the experience of the end user.

For readers familiar with technical usages of “black box,” (8, 10–14) our usage of *opaque* is similar with the exception it focuses on the experience of the end user and what they reasonably know or are presented with by a specific algorithm implementation. For readers familiar with the concept of “Explainable AI,” the “explainable” piece often refers to a secondary technology applied to a trained AI/ML model that extracts information about why the model makes certain predictions (6, 8, 11–15). An algorithm that makes use of such a technology and shows the output to an end user would be classified under our nomenclature as *translucent*.

## Methods and Definitions

The initial source of algorithms for consideration in this review is a constantly updated online database of FDA-approved algorithms. At the time of this writing, the database contained 70 such algorithms (1). As reported by the primary citation for the database (1), the majority of algorithms in this database were approved with 510(k) clearance. Other approval methods seen in the database are *de novo* pathway clearance and premarket approval clearance. The database makes broad categorizations of applicable fields for these algorithms. The fields most represented are Radiology, Cardiology, and Internal Medicine/General Practice.

Perioperative medicine is not explicitly mentioned in the database. Therefore, the categorization of “perioperative utility” in this review was made under the best judgment of the authors. The primary purpose of this work, though, is to establish a nomenclature, using the algorithms labeled as having perioperative utility in examples of applying this terminology. Of the 70 algorithms in the database, 22 were determined to have perioperative utility.

Each record in the database includes the name of the algorithm and the parent company. Using this information, along with the details in the corresponding FDA announcements themselves, journal reviewed articles describing the function of these algorithms were sought. This search included—but was not limited to—searching the parent company's website for mentions of journal articles. From this search, citations for 12 of the 22 algorithms were found.

The categories applied to these 12 algorithms were *opaque, translucent*, and *transparent*. Table 1 summarizes these categories. *Opaque* describes a system that (a) estimates non-linearly applied parameters that require advanced analysis (external to the product itself) to be understood, (b) estimates such a large set of parameters that a human cannot interpret them unless aided by a tool external to the product, or (c) provides a prediction with no indication as to the reason for the prediction. That is, an opaque system meets at least one of the criteria (a), (b), or (c). The general theme of this definition is whether the end consumer of the product's prediction also receives some measure of explanation as to why the specific prediction was made. Succinctly, if the user does not receive such an explanation, then the system is categorized as “opaque.”

**Table 1:** Summary of category definitions.

**Category and example usage**	**Defining features**
**Opaque** **Concrete example:** A clinician desires a prediction of future glucose values for a patient with no need to understand how the prediction was made	• Estimates non-linearly applied parameters that require advanced analysis to be understood • OR estimates such a large set of parameters that a human cannot interpret them unaided • OR provides a prediction with no indication as to the reason for the prediction
**Translucent** **Concrete example:** A clinician desires a prediction of future glucose values for a patient with an explanation of what clinical factors were involved in making the prediction	• Includes techniques for explaining the predictions from an otherwise opaque algorithm • Examples: • Plotting non-linear functions of features • Variable importance methods • Providing a list of factors considered
**Transparent** **Concrete example:** A clinician desires a prediction of future glucose values for a patient with an explanation of exactly how much each involved factor contributed to the prediction for each patient	• Estimates a linearly applied parameter or relatively small set of parameters that indicate how much each feature influences the output • OR indicates the specific rationale for a prediction • Example**:** providing the exact features and weights responsible for a given prediction • OR provides a prediction that is easily verifiable at the time of the prediction

*Translucent* describes a product that incorporates non-specific methods for assisting the end user in understanding possible reasons for a prediction that would otherwise be categorized as “opaque.” Examples include plotting non-linear functions of features, variable importance methods, and providing a list of factors considered. The general theme for the “translucent” category is that non-specific information about factors influencing the prediction is provided.

A system predicting diabetes diagnosis that considers weight, age and diet in its algorithm is translucent; if modified to provide the relative weights of each of these factors in making the diagnostic prediction, the algorithm would be considered transparent. The first case provides non-specific prediction factors (translucent) while the second includes specific information for the end-user (transparent). Similarly, for image recognition, placing the corresponding image (or wave form) next to a predicted label or highlighting a segment of an image corresponding to a prediction for image recognition would be *translucent*. Again, the distinction between *translucent* and *transparent* is non-specific vs. specific rationale for the prediction from the perspective of the end user. For comparison, the term *explainable* is used to describe tools used by a developer to make the output of a product more easily interpretable (16–18). While *translucent* conveys a similar idea, we emphasize that its definition is from the perspective of the end user and the kind of information a particular system based on an AI/ML algorithm provides to them. For an exploration of various meanings and uses of “Explainability” in the context of artificial intelligence and machine learning, see the work by Bhatt et al. (17).

*Transparent* describes a product that estimates a linearly applied parameter or relatively small set of parameters and is implemented to indicate to the user how much each feature influences the output or indicates the rationale for a prediction (e.g., providing exact weights for features considered). Alternatively, a transparent system's prediction is easily verifiable at the time the prediction is made using information the system provides. Consider the previous example of system that predicts a diabetes diagnosis and indicates BMI, age, and diet. This system is *translucent*, but an algorithm that provides the weights used for these features would be *transparent*.

## Examples of FDA-Approved Algorithms

Through the process described above, three of the 12 products with a located citations and clear perioperative utility were categorized as opaque (Table 2). These are RhythmAnalytics from Biofourmis Singapore Pte. Ltd., and the Guardian Connect System from Medtronic. RhythmAnalytics along with its underlying Biovitals Analytics Engine monitors, which categorizes cardiac arrhythmias via a convolutional neural network—a deep learning technique—that consumes wavelet transforms and short-time Fourier transforms of electrocardiogram (ECG) signals (19). Such a deep learning technique is inherently opaque, as the number of weights and their non-linear combination makes unaided understanding of the reasons for a prediction not feasible. The Guardian Connect System alerts users to interstitial glucose levels outside of a specified range. The system offers two alert types, “threshold” and “predictive.” The “threshold” alerts are transparent in that they indicate if the sensor glucose reading is above or below a threshold. The “predictive” alerts indicate whether glucose is predicted to be outside the specified range within the next 10–60 min (4). While the manufacturer's user guide (20) explains that these predictions are formula-based, prediction-based alerts are provided using the name of the prediction, not the reasons for the prediction. The categorizations presented here are based on the information provided to a user when a prediction is provided. Therefore, this system is opaque.

**Table 2:** System classifications.

**Name of device or algorithm**	**Online database description (1)**	**Description based on primary citation**	**Classification**
Biovitals analytics engine	“Cardiac monitor”	Detects prolonged QT interval (19)	Opaque
Rhythm analytics	“Monitoring cardiac arrhythmias”	Deep learning to classify rhythm (19)	Opaque
Guardian connect system	“Predicting blood glucose changes”	Predicts glucose levels outside of the normal range and gives predictive alerts (4, 20)	Opaque
eMurmer ID	“Heart murmur detection”	Determines if murmurs are innocent or pathologic (22)	Translucent
physIQ heart rhythm and respiratory module	“Detection of atrial fibrillation”	Uses patients' own baseline to detect changes (21)	Translucent
DreaMed	“Managing type 1 diabetes”	Recommends insulin doses (23, 24)	Translucent
ECG app	“Detection of atrial fibrillation”	Watch-based atrial fibrillation detection (25)	Transparent
FibriCheck	“Cardiac monitor”	Smartphone atrial fibrillation detection (26, 27)	Transparent
Irregular rhythm notification feature	“Detection of atrial fibrillation”	Smartphone irregular rhythm notification (28)	Transparent
WAVE clinical platform	“Monitoring vital signs”	Remote vital sign monitoring and alerts (29, 30)	Transparent
EchoMD automated ejection fraction software	“Echocardiogram analysis”	Helps place echo device. Calculates ejection fraction (31)	Transparent
Caption guidance	“Software to assist medical professionals in the acquisition of cardiac ultrasound images”	Works with EchoMD above (31)	Transparent

In the translucent category are products that provide a prediction alongside an upfront list of features considered—but no specific weights—or a non-specific visualization of signal used in making the prediction. Of the 12 systems, three fit the criteria for this category (Table 2). For example, the physIQ Heart Rhythm and Respiratory Module from physIQ Inc. collapses improving or declining factors related heart failure into a single index. The signals related to this index are viewable by patients and providers along with the corresponding calculated index (21), which led to categorizing this algorithm as translucent. Another cardiac monitoring product, eMurmer ID from CSD Labs GmbH, predicts whether murmurs are pathologic. Along with the prediction, the algorithm shows the systolic and diastolic phases considered; however, the specifics of the predictions “are protected under proprietary regulations,” (22) leading to inclusion of this algorithm in the translucent category. Likewise, the DreaMed algorithm from DreaMed Diabetes, Ltd., produces reports with important features related to its insulin dose recommendations (23, 24), placing it in the translucent category.

The remaining six (of 12) products fall in the transparent category (Table 2). Of these, three are implementations of atrial fibrillation detection that provide either annotated signals along with predictions in the case of ECG App from Apple Inc. (25) and FibriCheck from Qompium NV (26, 27) or use explicit “if… then…” rules in the case of the Irregular Rhythm Notification Feature from Apple Inc. (28). Similarly, the Wave Clinical Platform from Excel Medical Electronics LLC provides vital sign alerts based on a set of rules and provides the reason for the alert when triggered (29, 30). Also, in the transparent category is the EchoMD Automated Ejection Fraction Software which integrates with the Caption Guidance system from Caption Health Inc. to indicate when an a echocardiogram transducer is correctly placed for the automated calculation of ejection fraction (31). Since the reasons for the provided feedback are intuitively obvious (the transducer is or is not physically placed correctly), these systems are transparent.

### Additional Examples and Future Work

Here we have applied our proposed common language to FDA-approved algorithms, as initial examples of how these terms might be used in clinical contexts. We recognize there are many tools and devices in the world of perioperative medicine used for patient care, education, research, quality improvement, and operations. Covering all of these would be at least a book-length task, well beyond the scope of this project. However, we consider the next step in developing this nomenclature to be a review article addressing the (additional) most common algorithms for patient care. As a small sample, such a review article might include products such as BIS (Bispectral Index) (32, 33), Sedline (34), Datex-Ohmeda Entropy (35), and Edwards Hemosphere (36).

Beyond these additional examples in perioperative medicine, these terms are immediately extensible to other medical fields. While all examples provided herein dealt with algorithms surrounding surgery, note that the definitions themselves (Table 1) are agnostic to any medical subfield.

Additionally, some commentary seems warranted with respect to proprietary algorithms. Indeed, in this review, some products were given opaque or translucent classifications, which may change if the algorithmic details and/or source code for such products were ever released by the intellectual property owners. This dimension of the nomenclature whereby a products categorization could be changed by a public release of information further emphasizes that these terms are from the perspective of the end user rather than technical details.

## Conclusions

This work presents a nomenclature for describing algorithm implementations and applies it to several examples in the literature. This terminology is composed of three high-level categories: *transparent, translucent*, and *opaque*. These terms are applied the point of view of the clinician. To indicate how these terms can be used to categorize AI systems, AI/ML systems with FDA are presented as examples. A database of these examples that will be updated as new systems gain FDA approval is available at https://sites.uab.edu/periop-datascience/algo-database.

This nomenclature aids in understanding the appropriate use of models. In high-risk situation, the requirement for accuracy may be paramount. Alternatively, in high-profile situations, predictions may need to be explainable to stakeholders. For example, the FDIC in the United States requires financial institutions to develop “conceptually sound” (37) models. An assessment of conceptual soundness would be easiest for transparent models and most difficult for opaque models.

The primary values of common nomenclature are expediting and simplifying descriptions of model requirements and appropriate use between clinicians and developers.

## Author Contributions

RM performed the initial algorithm search and filtering and prepared the first manuscript draft. MB, ED, and SJ helped assess the perioperative utility of each algorithm and revised the manuscript. AS suggested the framework for the project, helped assess the appropriate category of each algorithm, and revised the manuscript. DB helped organize the project team, helped assess the perioperative utility of each algorithm, and revised the manuscript. All authors contributed to the article and approved the submitted version.

## Conflict of Interest

The authors declare that the research was conducted in the absence of any commercial or financial relationships that could be construed as a potential conflict of interest.

## Publisher's Note

All claims expressed in this article are solely those of the authors and do not necessarily represent those of their affiliated organizations, or those of the publisher, the editors and the reviewers. Any product that may be evaluated in this article, or claim that may be made by its manufacturer, is not guaranteed or endorsed by the publisher.
